# Supplementary material for: Expression of Bitter Taste Receptors in the Intestinal Cells of Non-Human Primates
Source: Int J Mol Sci. 2020 Jan 30;21(3):902. doi: 10.3390/ijms21030902 (PMC7037741; doi:10.3390/ijms21030902)
Supplement: Supplementary file 1 [file ijms-21-00902-s001.zip › upload/7045145-Supplementary Figures 0127.pdf]

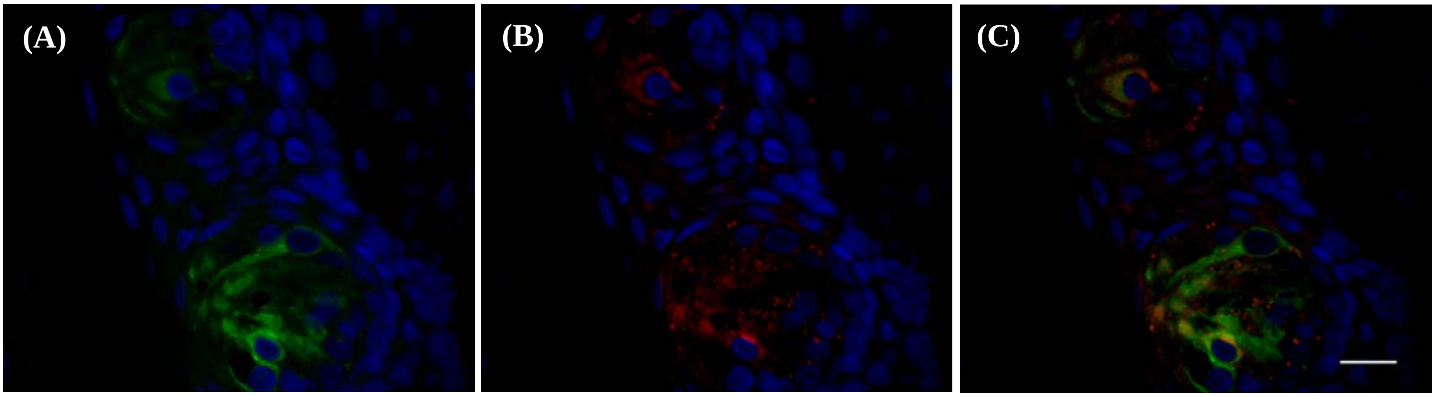

Figure.S1 Immunohistochemical detection of T2R38-positive cells in the CV. T2R38-positive cells were visualized by antibodies against T2R38 (green, A) and GNAT3 (red, B) with overlay. Nuclei were stained by DAPI (blue). Bar =10 µm.

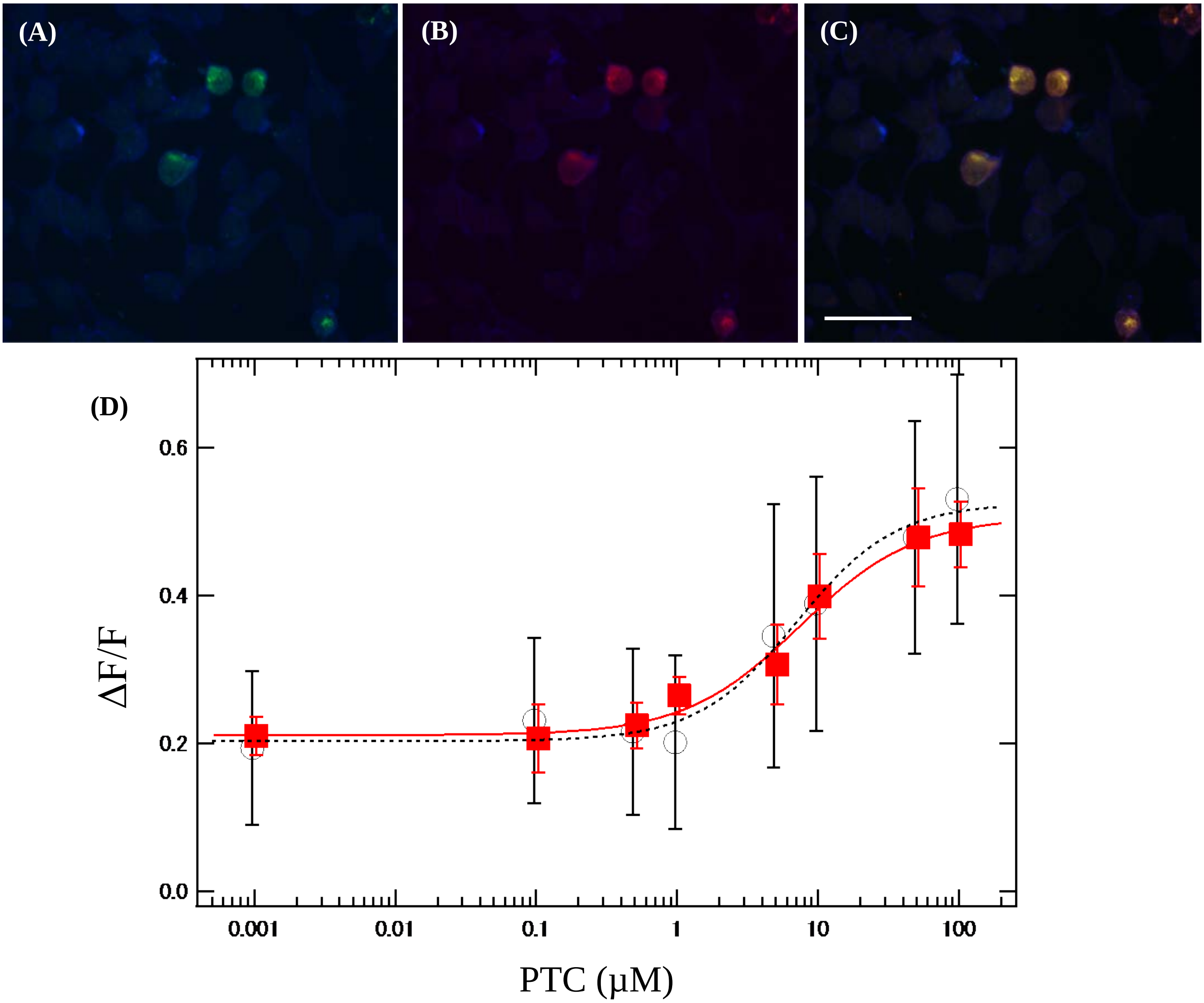

Figure S2 Immunocytochemical detection of T2R38 in HEK293 cells. HEK293 cells were transiently transfected with cDNA coding for the rhesus macaques taste receptor TAS2R38. T2R38-positive cells were visualized by antibodies against T2R38 (green, A) and 1D4-tag (red, B), with overlay (C). The cell surface was labeled with biotin-conjugated concanavalin A (blue). Bar = 50 μm. (D) Response to PTC from T2R38 transfected cells of human (circle) and macaque (square). The cells were transfected with the Gα16gust44 expression vector [15]. The calcium response amplitudes (ΔF/F) were defined as the ratio of the ligand-dependent increase in fluorescence to the fluorescence before ligand addition [5]. The average ΔF/F values from four independent experiments were plotted and a nonlinear regression function was fitted to the data as follows: f(x) = Imin + (Imax - Imin)/(1 + (x/EC50)h), where x is the ligand concentration and h is the Hill coefficient used to calculate EC50 (half maximal effective concentration) for the ligand–receptor interaction between PTC and TAS2R38 of human (dotted line, EC50=12.3 +/-7.7) and macaque (solid line, EC50=11.1 +/- 6.0)

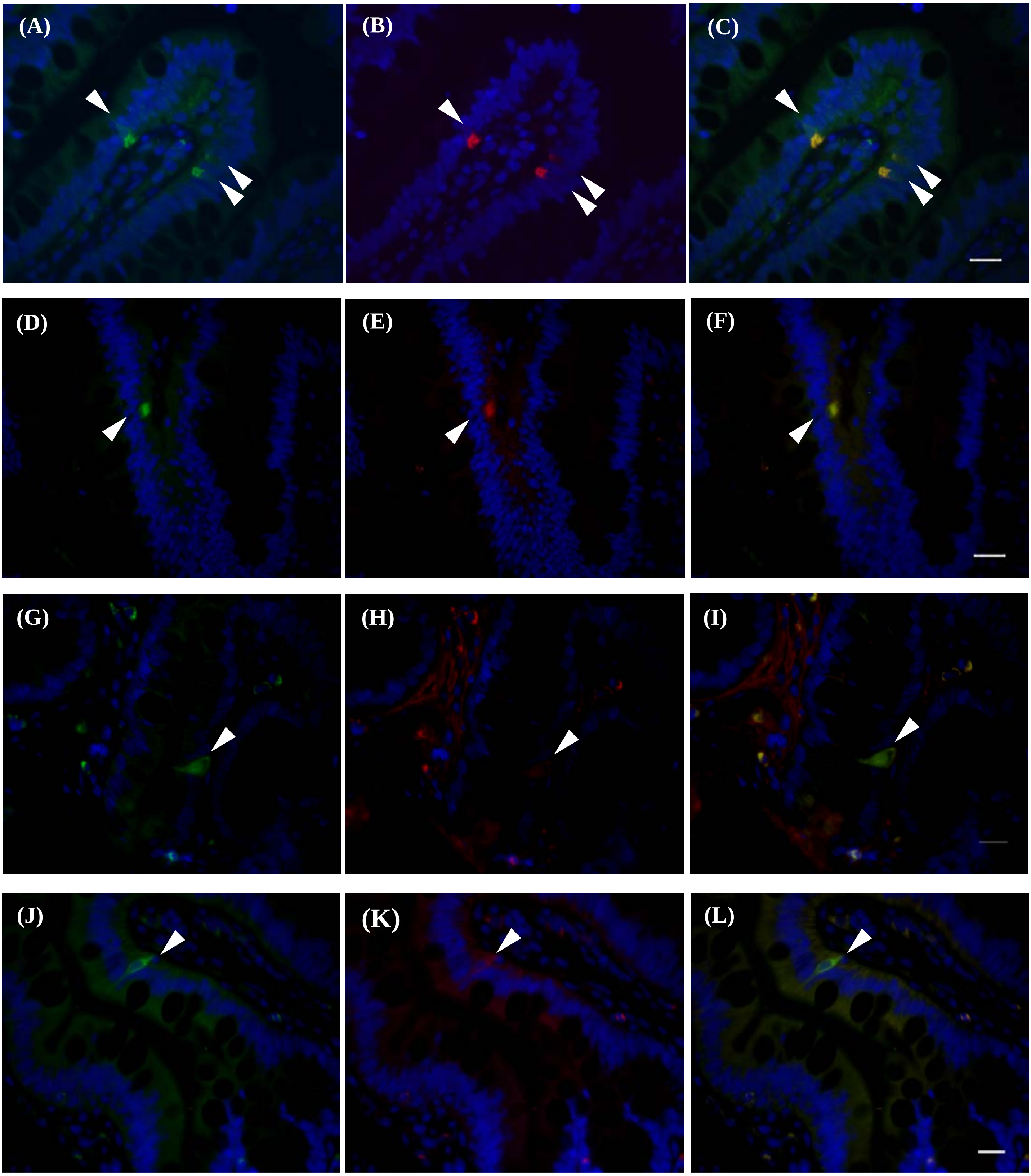

Figure S3 Immunohistochemical detection of T2R38-positive cells in the ileum. T2R38-positive cells were visualized by antibodies against T2R38 (green in A, D, G, J), chromogranin A (red in B), Mucin2 (red in E), DCLK1 (red in H), and GNAT3 (red in K) with overlay (C, F, H, L). Nuclei were stained by DAPI (blue). Arrowheads indicate the T2R38+/ marker + cells. Bar =10 μm.

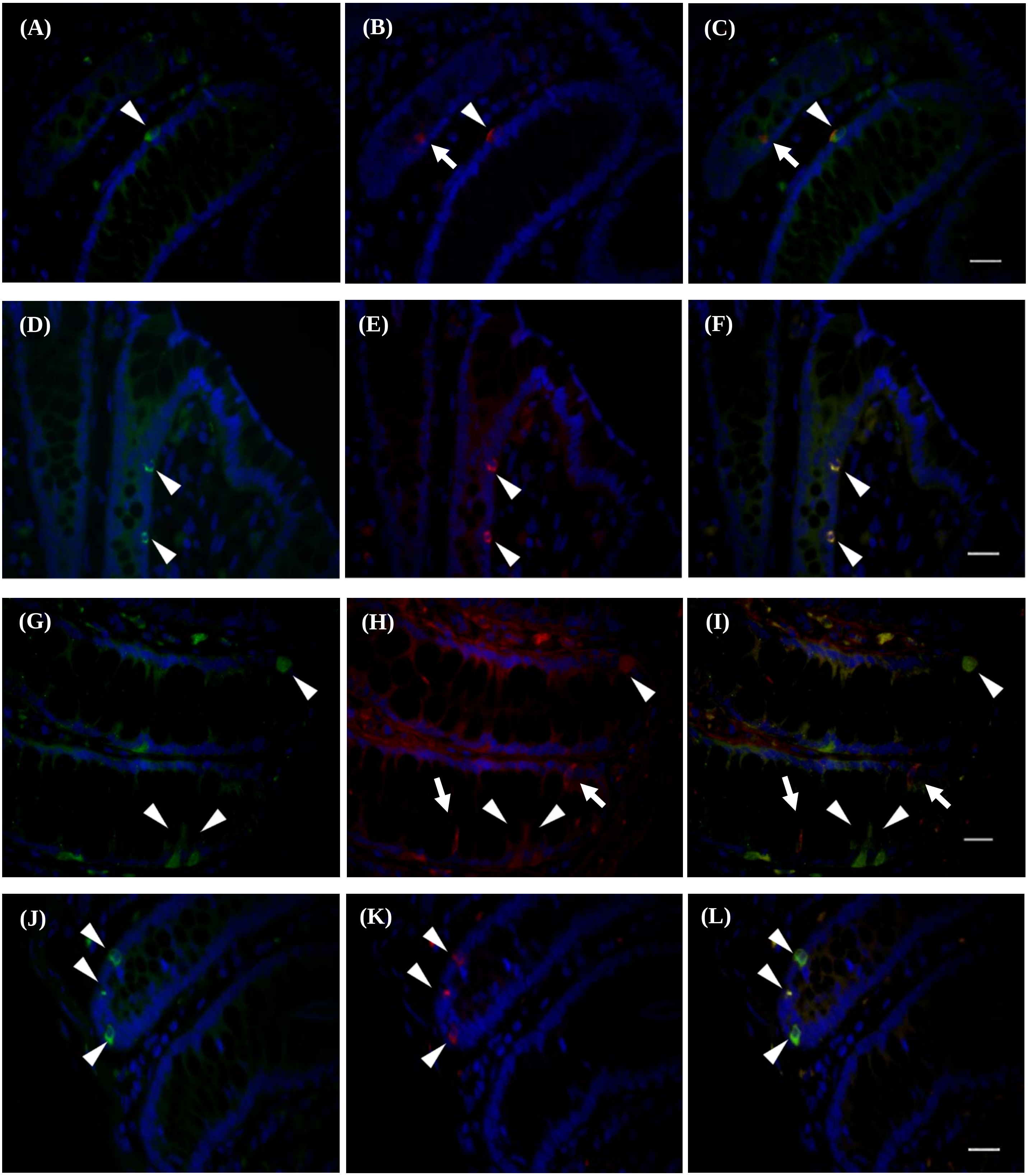

Figure.S4 Immunohistochemical detection of T2R38-positive cells in the colon. T2R38-positive cells were visualized by antibodies against T2R38 (green in A, D, G, J), chromogranin A (red in B), Mucin2 (red in E), DCLK1 (red in H), and GNAT3 (red in K) with overlay (C, F, H, L). Nuclei were stained by DAPI (blue). Arrowheads indicate the T2R38+/ marker + cells. Arrows indicate T2R38−/ marker + cells. Bar = 10 μm.
